# *In vivo* analysis of FANCD2 recruitment at meiotic DNA breaks in *Caenorhabditis elegans*

**DOI:** 10.1038/s41598-019-57096-1

**Published:** 2020-01-09

**Authors:** Marcello Germoglio, Anna Valenti, Ines Gallo, Chiara Forenza, Pamela Santonicola, Nicola Silva, Adele Adamo

**Affiliations:** 1 Institute of Biosciences and BioResources, National Research Council of Italy, Via Pietro Castellino 111, Naples, 80131 Italy; 2 Department of Biology, Faculty of Medicine, Masaryk University, Kamenice 5, 62500 Brno, Czech Republic

**Keywords:** DNA-binding proteins, Genomic instability, Gene regulation

## Abstract

Fanconi Anemia is a rare genetic disease associated with DNA repair defects, congenital abnormalities and infertility. Most of FA pathway is evolutionary conserved, allowing dissection and mechanistic studies in simpler model systems such as *Caenorhabditis elegans*. In the present study, we employed *C. elegans* to better understand the role of FA group D2 (FANCD2) protein *in vivo*, a key player in promoting genome stability. We report that localization of FCD-2/FANCD2 is dynamic during meiotic prophase I and requires its heterodimeric partner FNCI-1/FANCI. Strikingly, we found that FCD-2 recruitment depends on SPO-11-induced double-strand breaks (DSBs) but not RAD-51-mediated strand invasion. Furthermore, exposure to DNA damage-inducing agents boosts FCD-2 recruitment on the chromatin. Finally, analysis of genetic interaction between FCD-2 and BRC-1 (the *C. elegans* orthologue of mammalian BRCA1) supports a role for these proteins in different DSB repair pathways. Collectively, we showed a direct involvement of FCD-2 at DSBs and speculate on its function in driving meiotic DNA repair.

## Introduction

Fanconi Anaemia (FA) is a rare autosomal genetic disorder caused by mutations in any of the 22 complementation groups currently identified^1,2^ and is associated with progressive bone marrow failure (BMF), endocrine dysfunction, cancer, and other clinical features^3,4^.

FA pathway has been involved in repairing different types of damage, however it has been particularly linked to inter-strand cross-link (ICL) repair^5^. A large number of the FA proteins assemble into a complex (FANCA, B, C, E, F, G, L, M) which in response to DNA damage promotes ubiquitination of the ID2 complex, composed of FANCD2-FANCI, which in turn elicits repair by also functionally interacting with downstream factors, including BRCA1/2 and RAD51^6–13^. It has been previously shown that abrogation of the FA pathway leads to the accumulation of DNA double-strand breaks (DSBs) even under physiological conditions of growth^14^, suggesting that FA pathway is intrinsically required to maintain genome stability. Studies in different systems have shown that cellular defects associated with mutations in the FANCD2 can be greatly alleviated by elimination of Non-Homologous End Joining (NHEJ) factors, highlighting a role for FANCD2 in acting as a control switch between different DNA repair pathways^15–18^. FA proteins have been intensively studied during mitotic DNA repair and mostly in *ex vivo* systems, however their roles during gametogenesis have remained largely obscure, although some evidence suggests that these factors may be involved in promoting faithful chromosome segregation in meiosis. In particular, FANCD2 localizes on meiotic chromosomes in mouse spermatocytes^19^ and further, loss of FANCD2 induces defects in pairing and synapsis^20^. More recently, growing evidence highlighted a role for the FA pathway during meiotic DSB repair in a variety of organisms, providing new directions for research and diagnostics^21^.

*C. elegans* has emerged as a powerful model system to analyze DNA repair pathways *in vivo* and in particular, the gonad can be used as a toolkit for molecular and cellular analysis of DNA repair during meiosis, providing as a particularly amenable system for following the kinetics of formation and repair of DSBs. The *C. elegans* germline exhibits a time course of meiotic prophase I, in which nuclei in different stages can be easily identified based on their position and chromosome morphology.

During meiotic prophase I, DSBs are intentionally induced by topoisomerase-like SPO11 at meiosis onset, in order to allow recombination-dependent repair and the generation of crossovers (COs), which in turn promote equal segregation of chromosomes into the gametes. Given that DSBs carry a potential threat to genome integrity, the mechanisms that drive their repair must be tightly controlled. DSBs undergo end-resection, producing 3′ single-stranded DNA overhangs that initiate strand invasion into homologous sequence through RecA-like protein RAD-51, which forms discrete foci labeling all recombination intermediates. In most organisms, the number of DSBs is greater than final CO number and in *C. elegans*, only one DSB per homologue pair will result in a CO event^22^. The remaining DSBs are repaired through different, CO-independent mechanisms^23–26^.

Most key factors operating in the FA pathway are conserved between nematodes and humans^27^, making *C. elegans* a useful model to study genes driving human genetic disorders, in the context of a simpler organism. It has been previously shown that abrogation of *C. elegans fcd-2/FANCD2* function, a central player for functionality of FA pathway-mediated repair, induces hypersensitivity to ICLs-induced damage as observed in human derived cells, as well as developmental abnormalities, miss-regulation of CO formation and altered level/distribution of the RAD-51 recombination protein, suggesting a role of FCD-2 during meiosis^17,28–30^.

## Experimental Procedures

### Strains

Nematodes have been cultured at 20 °C on NGM plates with Escherichia coli OP50 as food source according to standard methods^31^.

The following *C. elegans* strains were provided by the *Caenorhabditis* Genetics Center:

*wild-type* strain Bristol N2; NB105 *fcd-2*(tm1298)IV; DW102 *brc-1 brd-1*(*tm1145- dw1)*III; NB120 *fnci-1*(*tm3081)*II.

The transgenic strain *3xFLAG::fcd-*2 was engineered by Knudra Transgenics. The strain AV106: *spo-11(ok79)/nT1[unc-119(n754)let-?(m435)GFPqls50*] IV;V was generated and provided by M. Colaiacovo; NSV159: *brc-1(ddr*2*8[GFP::brc-1])III*^32^. Deletions in the following mutants were genotyped during genetic crosses using PCR primers flanking the deletions listed in Table S1.

### CRISPR/Cas9-mediated genome editing

High efficiency clustered regularly interspaced short palindromic repeats-mediated gene editing was performed by Knudra Transgenics using proprietary vectors (Knudra Transgenics) to create the *3xFLAG::fcd-2 IV* strain. The transgene results from the insertion of three repeated sequences encoding a FLAG tag (GATTACAAGGATGACGATGACAAG), followed by a GSTGS linker in frame with the *fcd-2* coding region. Briefly, two plasmids expressing *fcd-2*-targeting guide RNAs (5′-AGATGATGAAAATAGTCATG-3′ and 5′-GCATCCCAACACGTGGAAGA-3′) were co-injected into 3 set of 10x N2 Bristol worms with a homology donor of 231 bp. A *dpy-10* sgRNA is added to DNA mix to create co-CRISPR dominant phenotype. F1 animals are screened for dominant roller phenotype.

At least one heterozygous positive is selected for homozygosing, as detected by PCR and screen for gene insertion.

### Proteins extraction from embryos

Worms were cultured at 20 °C on OP50 seeded NGM plates.

For embryos isolation, gravid adults were pelleted and treated with 30% NaOCl and 0.8 M NaOH for 12 min at room temperature, during which time the samples were vortex-mixed two or three times to resuspend and aerate the worms. Released embryos were collected in 0.1 M of NaCl and stored in liquid nitrogen.

For protein isolation, embryos were cracked by snap freezing in liquid nitrogen and then ground to a powder with a mortar and pestle. Samples were then solubilized adding an equal volume of 2X Lysis Buffer (100 mM HEPES; 2 mM EGTA; 2 mM MgCl2; 600 mM KCl; 20% Gycerol; 0.1% Nonidet P40; DTT 2 mM; Triton X 0.02%) containing protein inhibitors (Roche). Finally, total proteins were extracted by sonication followed by centrifugation at 13000 g for 10 min. to remove cellular debris^33^. The supernatant was divided in aliquots and stored at 80 °C for future use.

### Proteins extraction from worms

For whole protein extraction (from adults or age-mixed), 50 μL of worm pellet were resuspended in an equal volume of 2X Lysis Buffer containing 2X protein inhibitor (Roche). This suspension was snap-frozen in liquid nitrogen and thawed at 37 °C. The freeze-thawing was repeated twice before the addition of an equal volume of 2X Laemmli buffer^34^. Finally, samples were analyzed by SDS-PAGE.

### Western blot analysis

Protein extracts were prepared as described above and incubated for 5 min. at 100 °C. Different amounts of proteins were loaded on 4–20% gradient acrylamide gel and transferred onto PVDF membrane. After incubation with corresponding antibodies, the protein analysis was performed using ECL-PLUS kit (GE-Healtcare) as described before and VersaDoc apparatus (Bio-Rad) as previously described^35^. Primary antibodies used: Anti-FLAG -HRP conjugated antibody (abcam; 1:10000), Anti Actin (abcam 1:500) and Anti RAD-51 (1:1000).

### Viability screening

Worms were isolated and cloned during the L4 larval stage on Petri plates and left at 20 °C to lay eggs for 3 days. Worms were transferred every 12 hours onto fresh plates until the deposition of non-fertilized oocytes. Each plate was monitored every for 24/72 hours to analyze the following parameters among the progeny: embryonic lethality, presence of males and developmental defects. The percent of embryonic lethality is calculated as the ratio of unviable eggs on laid eggs. The percentage of males or developmental defects is calculated as the ratio of males/developmental defects on the hatched eggs^17,36,37^.

### DNA damage sensitivity

For cis-diamminedichloroplatinum(II) (CDDP) treatment synchronized animals were grown on OP50-seeded NGM plates in the presence of CDDP 180 µM. The worms were picked and transferred onto fresh plates (at the same concentration of CDDP) every 24 hrs for 72 hrs. The phenotypes were scored on progenies during the development. The laid eggs and dead embryos were scored after 0 hrs, 24 hrs and 48 hrs of treatment. For immunolocalization, the gonads were dissected immediately after 48 hrs of treatment. For western blot analysis, young adults were transferred on NGM plates containing CDDP. After 48 hrs of treatment the proteins were extracted from worms as described before.

For radiation treatment, adult worms were transferred onto freshly seeded plates and exposed to 120 Gy of γ-rays using a _137_Cs source and dissected and analysed after 1 hour^37^.

### Cytological analyses

Gonads or embryos from adults were dissected in M9 solution (0.3% H2PO4, 0.6% Na2HPO4, 0.5% NaCl and 1 mM MgSO4) on a Poly-Lysine glass slide. Embryos were fixed for 5 min in 2% paraformaldehyde. Slides were freeze-cracked in liquid nitrogen, then immersed at −20 °C in methanol, methanol/acetone (1:1) and acetone respectively for 5 min, followed by three washes in PBS for 5 min each time. Slides were blocked in 0.3% BSA in PBS for 30 min at 37 °C in a humid chamber. Primary antibodies used in this study were rabbit anti-RAD-51 and mouse anti-FLAG, both diluted 1:200 in Ab buffer (1% BSA, 0.1% Tween-20, 0.05% sodium azide in 1X PBS). Slides were incubated for 90 min at room temperature followed by three washes in PBS for 5 min. Appropriate secondary antibodies were Alexa-conjugated: goat anti-rabbit Texas Red and donkey anti-mouse Alexa Fluor 488 (1:400 dilution in Ab Buffer). Slides were incubated for 60 min in darkroom at room temperature, followed by three washes in PBS + 0.1% Tween-20 for 5 min each time. Slides were mounted with Prolong Gold Antifade reagent with 4′, 6′-diamidino-2-phenylindole hydrochloride (DAPI) (Life Technologies)^17,36,37^.

BRC-1::GFP fluorescence was directly observed as previously describes^32^. Gonads of young adult worms were dissected in PBS on a Polysine glass slide. After dissection slides were incubated on dry ice and then in ethanol at −20 °C for 1 min. Subsequently gonads were fixed by incubating slides with 2% paraformaldehyde in 0.1 M K_2_HPO_4_ (pH 7.2) for 10 min. at room temperature in a humid chamber. Slides were washed three times, 5 min. each time, in PBS + 0.1% Tween-20. After washes, each slide was incubated with 60 µL of 2 µg/ml solution of DAPI diluted in M9 for 10 min. at room temperature in a humid chamber in the dark. Slides were washed in PBST for 10 min. and then the presence of GFP fluorescence in late pachytene zone was detected.

### Analysis of DAPI-stained germlines

Adult nematodes were suspended in M9 solution on glass slides, permeabilized and fixed with 10 µl of 100% Et-OH. Finally, slides were mounted in 10 µl of DAPI (2 µg/ml) diluted in M9 solution. Numbers of scored nuclei are indicated in the legend of chart^17,37^.

### Quantitative analysis of germline apoptosis

Adult nematodes were suspended in M9 solution and stained by incubation with 33 µM SYTO-12 (Molecular probes) for 1 hr and 30 min at room temperature in the dark. The worms were then transferred to seeded plates to allow stained bacteria to be purged from the gut. After 30 minutes, the animals were mounted on 2% agarose pads in 2 mM levamisole. The estimation of apoptotic levels for each genotype was calculated as the average number of apoptotic nuclei per gonadal arm^17,37^.

### Image collection and processing

Collection of images was performed using a Leica DM6 fluorescence microscope, Hamamatsu camera under the control of Leica LAS X software. Images were processed and deconvolved using Leica LAS X software and Image J/Photoshop programs. Quantitative analysis of RAD-51 foci and DAPI-stained bodies along the germline were performed on z series, optical sections were collected at 0.18 µm and 0.50 µm increments respectively^36,37^. The images shown are maximum-intensity projections of z-stacks.

The quantitative analysis of RAD-51 and FLAG::FCD-2 foci were performed by dividing the germline into 7 zones (mitotic tip, mitotic zone, transition zone, early pachytene, middle pachytene, late pachytene stage and diplotene), according to chromosome morphology.

### Statistical tools

Statistical analyses for independent samples were computed through Mann-Whitney *t*-Student test. The level comparison of aberrant phenotypes of the different genotypes was computed through χ^2^ test. The Student’s t-test for independent samples was used for the analysis of apoptosis levels, RAD-51 foci, diakinesis, and western blot.

## Results

### Construction and characterization of a new *C. elegans* strain containing a functional FLAG-tagged FCD-2

Taking advantage of the attributes of the *C. elegans* system, ease of genome editing, and the spatio-temporal organization of the germ line, we have obtained a strain containing tagged FCD-2 and then analyzed the protein localization and function. By CRISPR/Cas9 genome-editing technology, we introduced a sequence of three repeated FLAG at the 5′ of the endogenous *fcd-2* locus (see Mat and Met for details). The presence of FLAG allowed us to perform *in vitro* and *in vivo* studies of FCD-2 using an antibody direct against the tag.

**Figure 1:**
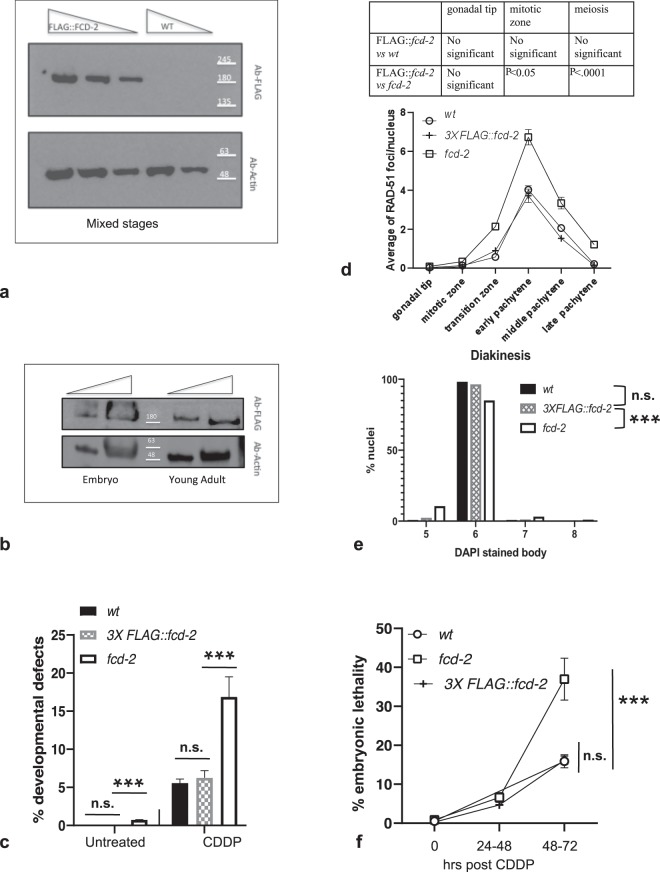
Analysis of *FLAG::fcd-2* transgenic strain. (**a**) Western blot analysis of protein extracts prepared from mixed stages of development of WT or *FLAG-fcd-2* strains. (**b**) Western blot analysis of protein extracts from embryos and young adult *FLAG::fcd-2* worms. The filters were probed with antibodies against the indicated protein. The panels (**a**,**b**) show different parts of the same filter probed with indicated antibodies. Anti -Actin was used as loading control. The standard molecular weight marker was showed. (**c**) Percentage of developmental defects/ hatched eggs in indicated genotypes observed before and after 24–48 hrs treatment with 180 μM CDDP. Number of the developmental defects/hatched eggs observed: *wt* (N2) untreated = 0/2480; *fcd-2* untreated = 15/2018; *FLAG::fcd-2* untreated = 1/3593; *wt* (N2) CDDP = 50/866; *fcd-2* CDDP = 89/526; *FLAG::fcd-2* CDDP = 72/1169. Developmental defect phenotypes observed: dumpy, long, multivulva, roller, small, uncoordinated, larval arrest, vulvaless, vulva protrusion, bag of worm. Error bars correspond to SD calculated from at least three independent experiments. P value (x^2^ test): n.s. = no significant difference; ***P < 0.0001. (**d**) Quantification of RAD-51 foci in the indicated genotypes. The y-axis represents the average of RAD-51 foci per nucleus. The x-axis represents the position (zone) along the germline. An average of 90 nuclei for each gonad region were scored for each genotype. Error bars correspond to SEM calculated from at least three independent experiments. In table are indicated the significant differences obtained by the Student’s t-test for independent samples. (**e**) Quantification of DAPI stained body in diakinesis nuclei in indicated genotypes. Number of diakinesis nuclei scored: *wt* (N2) = 114; *FLAG::fcd-2* = 84; *fcd-2* = 94. P value (x^2^ test): n.s. = no significant difference; ***P < 0.0001. (**f**) Embryonic lethality at different time points after treatment with 180 μM CDDP for indicated genotypes. Error bars correspond to SEM calculated from at least three independent experiments. P value (48–72 hrs) (x^2^ test): n.s. = no significant difference; ***P < 0.0001.

First, we analyzed the obtained strain for any potential defects due to loss of functionality of the fusion protein. To be sure that the presence of the tag did not affect expression of FCD-2, we performed western blot on worm extracts at mixed stages of development. As shown in Fig. 1a, the anti-FLAG antibody specifically recognizes a single band at the expected molecular weight of full-length FCD-2 in the *FLAG::fcd-2* strain but not in the wild type (*wt*), indicating that the fusion protein is correctly expressed. We also wondered whether FCD-2 was expressed at specific developmental stage. We then performed western blot analysis on protein extracts from embryos and young adult animals and found that the protein was constitutively expressed (Fig. 1b).

We also screened the *FLAG::fcd-2* strain and found that viability and brood size were similar to the wild type animals and importantly, no segregation of worms carrying developmental defects was observed (Table 1, Fig. 1c). The distinctive phenotypes of *fcd*-2 mutant, such as altered level/distribution of RAD-51 foci during meiosis (Fig. 1d), miss-regulation of crossing-over formation (Fig. 1e) and apoptosis (data not shown) were not found. Moreover, unlike *fcd-2* mutants, *FLAG::fcd-2* animals are not hypersensitive to the ICL-inducing agent *cis*-platin (CDDP), showing a frequency of embryonic lethality after CDDP treatment (48–72 hrs) similar to wild type animals (Fig. 1f), as well as the frequency of developmental defects before and after CDDP treatment (24–48 hrs) (Fig. 1c). Taken together the results indicate the full functionality of the FLAG::FCD-2 protein.

**Table 1:** Screening of wild type (*wt*), *fcd-2* and *3xFLAG::fcd-2*.

Strains	*wt*	*fcd-2*	*3X FLAG::fcd-2*
Parentals	10	9	12
laid eggs	2493	2035	3603
dead embryos	13	17	10
hatched eggs	2480	2018	3593
brood size	249.3	226.1	300.25
developm.defects	0	15	1
% dead embryos/ laid eggs	0.52	0.84	0.28
% developm.defects/ hatched eggs	0.0	0.74	0.028

Developmental defect phenotypes observed: dumpy, long, multivulva, roller, small, uncoordinated, larval arrest, vulvaless, vulva protrusion, bag of worm.

*X*^*2*^ test developmental defects: wild type (*wt*) vs *3XFLAG:: fcd-2:* P = 0.86.

### Analysis of FCD-2 localization in the germline

**Figure 2:**
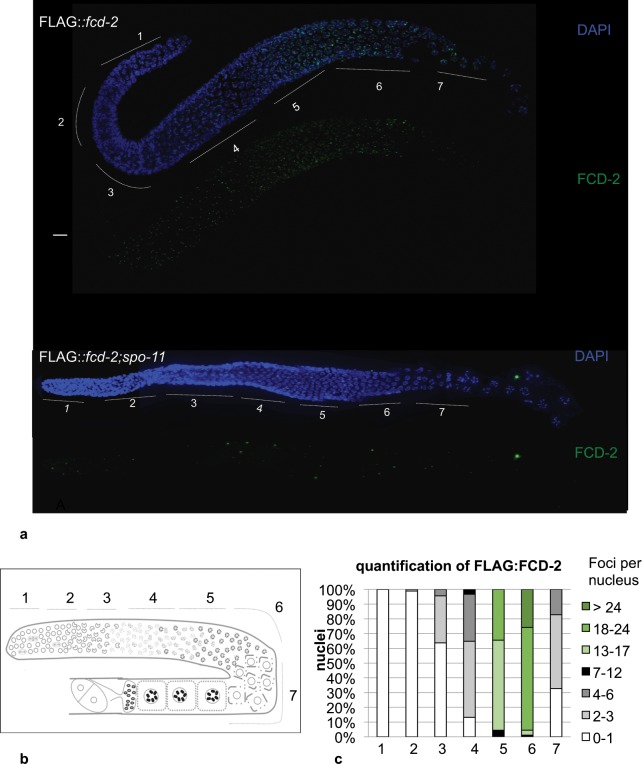
Localization of FLAG::FCD-2 in the *C. elegans* germline. (**a**) Dissected gonads from *FLAG::fcd-2* and *FLAG::fcd-2 spo-11* transgenic hermaphrodite worms were co- stained with anti-FLAG antibody (green) and DAPI (blue). No signal was observed in absence of *spo-11* gene. Bar, 10 μm. (**b**) Schematic representation of the *C. elegans* gonad arm. Regions 1 and 2 consist of mitotic nuclei, region 3 consists of meiotic nuclei in transition zone (leptotene and zygotene). The regions 4, 5 and 6 correspond to early, middle and late pachytene respectively. The region 7 consists of diplotene nuclei. (**c**) Histograms represent quantification of FLAG::FCD-2 foci in germline of *FLAG::fcd-2* strain. The y-axis represents the percentage of nuclei with the indicated number of foci. The x-axis represents the position (zone) along the germline.

We then sought to investigate expression and loading dynamics of FCD-2 in the germline, as its localization during gametogenesis is not known. A standard approach to evaluate the *C. elegans* germline involves dividing the gonad into different zones, followed by quantitative analysis of meiotic events in each of these zones through employment of specific markers^38^. Zones 1 and 2 consist of mitotic nuclei (gonad tip and mitotic zone) undergoing proliferation to generate the nuclei that enter meiotic prophase through the “transition zone” (zone 3) in which chromosomes acquire a polarized organization. At pachytene stage (zones 4–6) chromosomes disperse from polarized organization and the synaptonemal complex becomes linear and completely formed. Toward the end of pachytene (zones 6) some nuclei undergo physiological apoptosis^39^, while other progress into diplotene (zone 7) and then in diakinesis stage, differentiating into oocytes (Fig. 2a,b).

Immunofluorescence analysis was performed as described in material and methods, washing out weakly bound proteins and leaving only the ones stably bound to chromatin. Anti-FLAG antibody reveals that FCD-2 is absent in the most of the premeiotic nuclei (gonad tip and mitotic zone) and is loaded on chromosomes at the onset of meiosis (zone 3–4), with an upward trend up to the late pachytene stage (zone 6) and declined at diplotene stage (zone 7). Interestingly, more then 90% of nuclei in middle (zone 5) and late pachytene (zone 6) stages contained at least 13 foci/nucleus (Fig. 2c). We also verified the presence of FCD-2 on chromosome in the embryos, however no foci were detected (Figure S1a). Given its localization, it is conceivable to envision that FCD-2 might exert predominant roles during meiosis under unchallenged conditions of growth, in particular at pachytene stage, where its recruitment along the chromosomes displays higher enrichment.

### FCD-2 loading requires double-strand breaks formation but is independent of RAD-51

**Figure 3:**
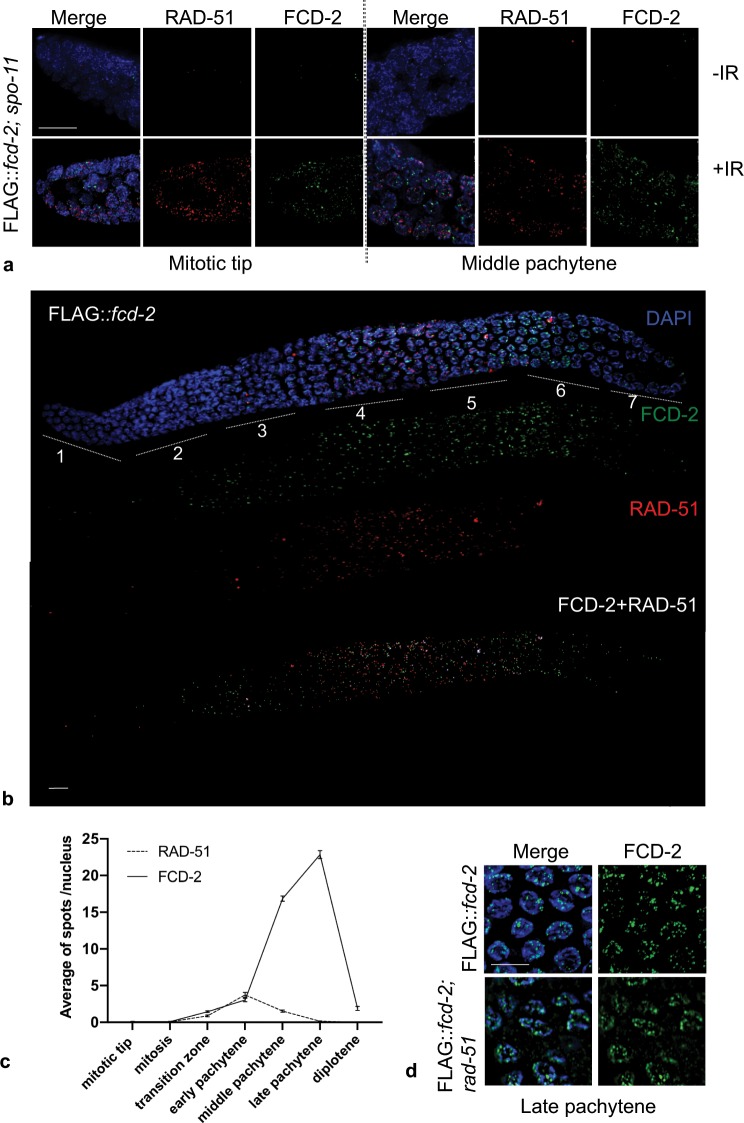
FCD-2 localization along germline requires DSBs but not RAD-51. (**a**) Representative images of the Mitotic tip and Middle pachytene nuclei in *FLAG::fcd-2 spo-11* transgenic hermaphrodites co-stained with anti-FLAG antibody (green), anti-RAD-51 (in red) and DAPI (blue), before and after (1 hour) 120 Gy γ-rays exposure. Bar, 10 μm. (**b**) Representative image of dissected gonad from *FLAG::fcd-2* transgenic hermaphrodite worm and co-stained with anti-FLAG antibody (green), anti-RAD-51 (in red) and DAPI (blue). Overlapping signal is shown in the white areas. Regions 1 and 2 consist of mitotic nuclei, region 3 consists of meiotic nuclei in transition zone (leptotene and zygotene). The regions 4, 5 and 6 correspond to early, middle and late pachytene respectively. The region 7 consists of diplotene nuclei. Bar, 10 μm. (**c**) Quantification of FLAG::FCD-2 foci and RAD-51 foci in germline of *FLAG::fcd-2* strain. The y-axis represents the average of foci per nucleus. The x-axis represents the position (zone) along the germline. An average of 90 nuclei for each gonad region were scored for each genotype. Error bars correspond to SEM calculated from at least three independent experiments. (**d**) Representative images of late pachytene nuclei in *FLAG::fcd-2* and *FLAG::fcd-2 rad-51* worms co-stained with anti-FLAG antibody (green) and DAPI (blue). Bar, 10 μm.

Successful completion of meiosis requires the induction and processing of DSBs. These are physiologically produced by SPO-11, followed by resection and invasion of the homologous chromosome to accomplish repair, which is mediated by RAD-51 protein^37,40–42^. The absence of *spo-11* gene in *C. elegans* prevents the formation of DSBs and consequently the recruitment of DNA strand-exchange protein RAD-51 at DNA breaks^43^. To establish whether FCD-2 loading depends on DSBs and/or recombination intermediates, we generated a *FLAG::fcd-2; spo-11* double mutant and analyzed FCD-2 localization. As expected, RAD-51 loading was impaired in *FLAG::fcd-2; spo-11* doubles in line with lack of DSBs (Fig. 3a). Strikingly, impaired formation of DSBs disrupted loading of FCD-2 as well (Figs. 2a and 3a) suggesting a putative function at meiotic DSBs. We then decided to evaluate whether exogenous DSBs could restore FCD-2 localization in the germline or rather SPO-11 having a direct involvement in FCD-2 loading. To test this hypothesis, the *FLAG::fcd-2; spo-11* strain was exposed to γ-irradiation and the presence of DSBs was verified evaluating the distribution of RAD-51 along the gonad (Fig. 3a). Interestingly, DNA damage induced recruitment of FCD-2 in both premeiotic and meiotic nuclei (Fig. 3a), indicating that the presence of the protein on chromosomes depends on DSBs, regardless if they are physiologically induced or generated by ectopic damage. Since *spo-11* deletion impairs DSBs formation but also RAD-51 loading, we co-stained FCD-2 and RAD-51, as well as analyzed FCD-2 loading in *rad-51* mutants to assess whether FCD-2 loading required DSBs or RAD-51. As shown in Fig. 3b,c, RAD-51 and FCD-2 display different expression kinetics in the germline and do not display a clear co-localization. Furthermore, FCD-2 localization did not change in absence of RAD-51 indicating that FCD-2 recruitment to chromosomes does not depends on the presence/activity of RAD-51 (Fig. 3d). Collectively, these data suggest that FCD-2 protein is required at DSB in a RAD-51 independent manner.

### Genotoxic insults upregulate FCD-2 recruitment in the germline

In mammalian cells, the FA pathway participates in the initial stage of ICL repair, which involves the recognition of ICL and their conversion to DSBs. Previous data in *C. elegans* demonstrated that *fcd-2* mutant is hypersensitive to ICLs^17,29^, Fig. 1c–f), however the effect of ICL damages on protein expression/stability is still unknown.

**Figure 4:**
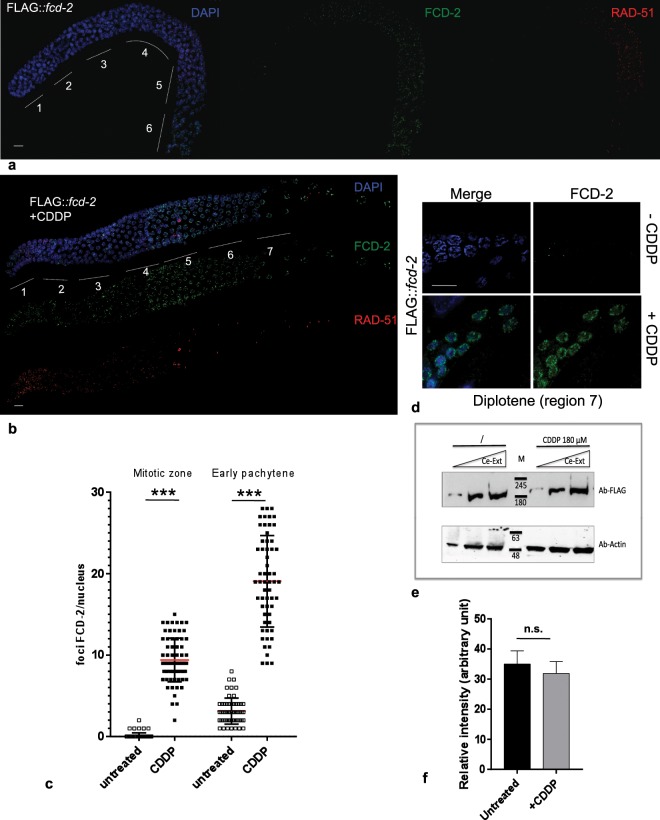
Localization and expression of FLAG::FCD-2 after ICL damage in the *C. elegans* germline. (**a**) Representative image of gonad dissected from *FLAG::fcd-2* transgenic hermaphrodite worm and co-stained with anti-FLAG antibody (green), anti-RAD-51 (in red) and DAPI (blue). Bar, 10 μm. (**b**) Representative image of gonad dissected from *FLAG::fcd-2* transgenic hermaphrodite worm and co-stained with anti-FLAG antibody (green), anti-RAD-51 (in red) and DAPI (blue) after 48 hours CDDP treatment. Bar, 10 μm. Regions 1 and 2 consist of mitotic nuclei, region 3 consists of meiotic nuclei in transition zone (leptotene and zygotene). The regions 4, 5 and 6 correspond to early, middle and late pachytene respectively. The region 7 consists of diplotene nuclei. (**c**) Strip chart of FCD-2 foci in mitotic zone and early pachytene of *FLAG::fcd-2* strain before and after 48 hours CDDP treatment. Each dot indicates the number of FCD-2 foci per nucleus. Red bar indicates the average for each different condition. Error bars correspond to SD calculated from at least three independent experiments. P value (Student’s t-test): ***P < 0.0001. (**d**) Representative images of diplotene nuclei in *FLAG::fcd-2* transgenic hermaphrodites co-stained with anti-FLAG antibody (green), and DAPI (blue), untreated or treated with CDDP. Bar, 10 μm. (**e**) Western blot analysis of increasing amounts of protein extracts prepared from synchronized worms (*FLAG::fcd-2* strain) treated or not with CDDP. The same filter was probed with antibodies against the indicated proteins. The panel shows different parts of the same filter probed with indicated antibodies. Anti -Actin was used as loading control. Data presented are representative of at least three independent experiments performed with at least three different amount of Protein extract. (**f**) Quantitation of FCD-2 levels normalized against Actin. Signal intensity of each band was measured using Quantity One software (Biorad). The y-axis represents the relative expression of FCD-2 in protein extracts from untreated worms and after CDDP treatment. Bars represent the means ± error standard from at least three independent experiments performed with three different amount of Protein extract for each experiment. P value (Student’s t-test): n.s. = no significant difference.

In order to investigate FCD-2 localization after ICL damages, we performed *in vivo* analysis of worms treated with CDDP. As shown in Fig. 4 and Table S2, the number and the distribution of FCD-2 foci stably bound to chromatin observed in the germline were dramatically different in the treated worms compared to the control (Fig. 4a,b). In particular, analysis of FCD-2 foci in the different zones along the gonad after CDDP treatment showed that: i) the protein increases in the premeiotic nuclei (p < 0.0001) (Fig. 4c, Table S2); ii) the number of foci in the meiotic nuclei was strongly increased (p < 0.0001) (Fig. 4c, Table S2); and iii) the foci persisted at diplotene stage (Fig. 4d). We also examined the embryonic cells showing that FCD-2 localized on chromosome in response to ICL damages (Figure S1b). Similar results were found after exposure to γ -irradiation (Figures S1c, S2).

In order to determine the expression levels of protein after CDDP treatment we analyzed by western blot of whole worm extracts before and after DNA damage. Surprisingly, the treatment with CDDP did not influence FCD-2 expression levels (Fig. 4e,f).

Collectively, these data demonstrate that genotoxic insults do not affect the stability and the expression of FCD-2 but induce protein recruitment at sites of damage, where most likely FCD-2 is required to augment DNA repair.

### Loading of FCD-2 depends on functional FNCI-1 protein

**Figure 5:**
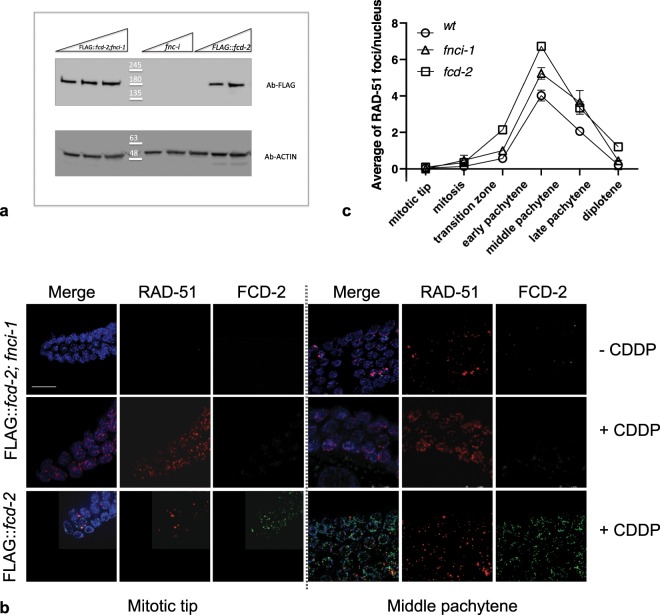
Loading of FCD-2 on chromosomes is FNCI-1-dependent. (**a**) Western blot analysis of increasing amounts of protein extracts prepared from *FLAG::fcd-2; fcni-1*, *fnci-1*, *FLAG::fcd-2* strains. The same filter was probed with antibodies against the indicated proteins. The panel shows different parts of the same filter probed with indicated antibodies. Anti -Actin was used as loading control. Data presented are representative of at least three independent experiments performed. (**b**) Representative images of the Mitotic tip and Middle pachytene nuclei in indicated genotypes co-stained with anti-FLAG antibody (green), anti-RAD-51 (in red) and DAPI (blue), under indicated conditions. Bar, 10 μm. (**c**) Quantification of RAD-51 foci in the indicated genotypes. The y-axis represents the average of RAD-51 foci per nucleus. The x-axis represents the position (zone) along the germline. An average of 90 nuclei for each gonad region were scored for each genotype. Error bars correspond to SEM calculated from at least three independent experiments.

Since human FANCI and FANCD2 form a heterodimer named ID2 complex^44^, we investigated whether FCD-2 loading onto chromosomes is dependent on the presence of FNCI-1^45^, (orthologue of human FANCI). We generated the *fnci-1; FLAG::fcd-2* strain and analyzed the expression levels and distribution of FCD-2. Western blot analysis of worm extracts at mixed stages showed that absence of FNCI-1 did not affect the total amount and stability of FCD-2 (Fig. 5a). Surprisingly, *in vivo* analysis showed that loading of FCD-2 in the germline of *fnci-1* mutants was completely abolished (Fig. 5b). To exclude that absence of FCD-2 foci was due to defects in DSB formation in *fnci-1* mutants, we analyzed the level/ distribution of RAD-51. However, *fnci-1* mutants show a meiotic RAD-51 regular distribution, indicating the presence of double strand breaks (Fig. 5c). Similar results were obtained after treatment with DNA damage-inducing agent, showing lack of FCD-2 foci in absence of a functional FNCI-1 both in the germline and embryos (Figs. 5b and S1d–f). Collectively, these data show that FNCI-1 does not influence FCD-2 expression levels but is essential for its loading at DSBs at all developmental stages.

### Functional interaction between FCD-2 and BRC-1 in meiosis

Breast and ovarian cancer susceptibility protein BRCA1 and its heterodimeric partner BARD1 play a fundamental role in DNA repair in mitotic cells. Kais and collaborators demonstrated that FANCD2 cooperate with BRCA1/2 proteins to maintain genomic integrity through replication fork stabilization. Moreover, they showed that the loss of FANCD2 in BRCA1/2-deficient tumors enhances cell death, suggesting a synthetic lethal relationship between FANCD2 and BRCA1/2^46^. Important studies obtained in *C. elegans* reported that BRC-1 and its partner BRD-1 localize to chromosomes at all stages of meiotic prophase I, highlighting a role in DSB repair during gametogenesis^32^, however the relationship between FANCD2 and BRCA1/BARD1 is not well known. Thus, we investigated the functional interaction between the two proteins by exploiting the *C. elegans* germline system.

**Figure 6:**
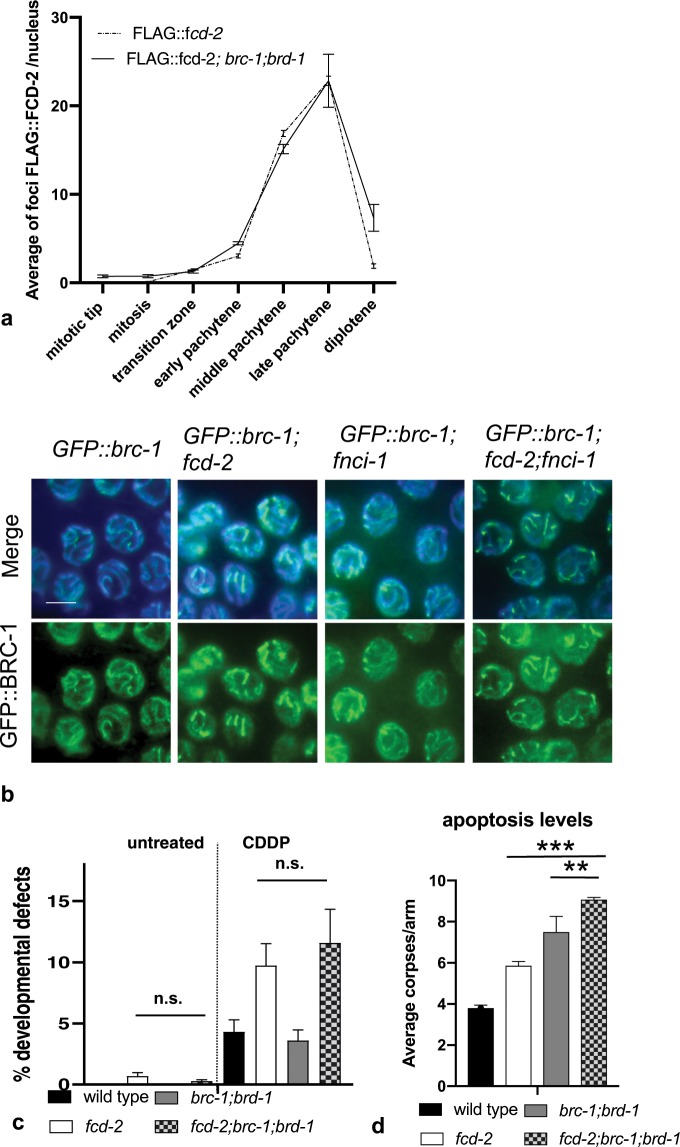
Genetic interaction between FCD-2 and the BRC-1/BRD-1 complex. (**a**) Quantification of FLAG::FCD-2 foci in germline of *FLAG::fcd-2* and *FLAG::fcd-2; brc-1 brd-1* strains. The y-axis represents the average of FLAG::FCD-2 foci per nucleus. The x-axis represents the position (zone) along the germline. An average of 60 nuclei for each gonad region were scored for each genotype. Error bars correspond to SEM calculated from at least three independent experiments. (**b**) Representative images of the late pachytene nuclei in indicated genotypes co-stained with anti-GFP antibody (green), and DAPI (blue). Bar, 5μm. (**c**) Graphic representation of the percentage of developmental defects/hatched eggs in the indicated genotypes observed before and after 24–48 hrs treatment with 180 μM CDDP. Number of the developmental defects/hatched eggs observed: *wt* (N2) untreated = 0/2480; *fcd-2* untreated = 15/2018; *brc-1 bard-1* untreated = 0/3972; *fcd-2; brc-1 bard-1* untreated = 5/1810; *wt* (N2) CDDP = 30/696; *fcd-2* CDDP = 60/617; *brc-1 bard-1* CDDP = 50/1390; *fcd-2; brc-1 bard-1* CDDP = 38/328. Developmental defect phenotypes observed: dumpy, long, multivulva, roller, small, uncoordinated, larval arrest, vulvaless, vulva protrusion, bag of worm. Error bars correspond to SD calculated from at least three independent experiments. P value (x^2^ test): n.s. = no significant difference. (**d**) Quantification of germline apoptosis in the indicated genotypes. The y-axis shows the average number of SYTO-12-labeled nuclei per gonadal arm. Number of observed gonad: wt: 84; *fcd-2*: 109*; brc-1 brd-1*: 73*; fcd-2; brc-1 brd-1*: 74. Error bars correspond to SD calculated from at least three independent experiments. P value (Student’s t-test): **P < 0.05; ***P < 0.0001.

We first wondered whether loading of BRC-1 and FCD-2 on chromosomes during meiosis is mutually dependent. To this end, we built the *brc-1 brd-1; FLAG::fcd-2* mutant strain and analyzed FCD-2 foci distribution along the germline. Quantitative analysis of foci in the different zones along the gonad showed that the absence of BRC-1/BRD-1 complex does not influence FCD-2 distribution (Fig. 6a). Then we investigated the ability of BRC-1 to load on chromosome in absence of one or both component of FCD-2/FNCI-1 heterodimer demonstrating that BRC-1 localized at late pachytene, similar to the wild type (Fig. 6b). Collectively the data suggest that either FCD-2 and FNCI-1 are dispensable for BRC- 1 loading. We finally wondered whether the simultaneous absence of *fcd-2* and *brc-1 brd-1* influences worms fertility, and analysis of the triple mutant showed that viability and segregation of worms with developmental defects were similar to the *fcd-2* mutant under both physiological conditions (Fig. 6c and Table 2) and after ICL induction (Fig. 6c). Furthermore, since *fcd-2* and *brc-1 brd-1* mutants show increased levels of physiological apoptosis^17,47,48^, we evaluated impact on this process in absence of both proteins. Interestingly, we found that the average number of apoptotic pachytene nuclei was further increased in *brc-1 brd-1; fcd-2* triple mutant (Fig. 6d).

**Table 2:** Screening of *brc-1 brd-1*, *fcd-2* and *fcd-2; brc-1 brd-1*.

Strains	*brc-1 brd-1*	*fcd-2*	*fcd-2; brc-1 brd-1*
parentals	23	9	14
laid eggs	4002	2035	1820
dead embryos	30	17	10
hatched eggs	3972	2018	1810
developm.defects	0	15	5
% dead embryos/laid eggs	0.75	0.84	0.55
% developm.defects/ hatched eggs	0	0.74	0.28

Developmental defect phenotypes observed: dumpy, long, multivulva, roller, small, uncoordinated, larval arrest, vulvaless, vulva protrusion, bag of worm.

*X*^*2*^ test developmental defects: *fcd-2* vs *fcd-2; brc-1 brd-1:* P = 0.075.

It is reported that in *C. elegans*, accumulation of unrepaired DNA damage in the germ line either in response to genotoxic stress or upon failure to faithfully execute meiotic recombination induces apoptotic cell death^49^. In this view, our data support a role for FCD-2 and BRC-1 in the repair of DNA damage likely driving different DNA repair pathways.

## Discussion

Genomic instability is a hallmark of cancer and promotes the acquisition of genetic aberrations that ultimately favor oncogenic transformation^50^. Understanding the molecular mechanisms of DNA damage response is hence essential for advancing cancer research. Due to the considerable importance of the FA network in maintaining genome integrity, a large body of research has been directed to this pathway. The FA pathway plays a central role in ICL repair, during which the FA proteins coordinate the balance between NHEJ and homologous recombination repair ensuring genome stability. Besides in the canonical ICL repair functions, emerging view suggests that the FA pathway plays a key role in genome stability maintenance. In particular, FA proteins protect against aberrant chromosomal structure and replication stress by interacting with factors involved in recombination and repair^51,52^. FANCD2 is a key player in FA pathway and is detectable at ICL^53^ and UV-induced damage^54^, however the biological significance of FANCD2 recruitment is still unknown. Recent results in cell lines demonstrated that FANCD2 localizes at DSBs induced by nuclease Cas-9, indicating a direct role in regulating genome editing^16^.

By employing an amino-terminal tag of *Ce*FANCD2, we showed *in vivo* that FCD-2 localization is dynamic during prophase I and requires its partner FANCI-1 to load onto DNA, raising the possibility that the proteins work together to protect genome integrity during meiosis. Strikingly, SPO-11 activity is essential for FCD-2 recruitment at chromosomes, which instead does not require RAD-51-dependent formation of recombination intermediates, suggesting a putative localization at DSBs.

It is known that DSB repair activity is strongly controlled such that only one DSB per chromosome pair is repaired via crossover recombination; whereas the other DSBs are repaired by different pathway^22^. In early pachytene, where RAD-51 is more loading, DSBs are repaired as inter-homolog crossovers RAD-51-dipendent. FCD-2 protein stably bound to DNA is detected on chromosomes at the onset of meiosis with an upward trend up to the late pachytene, where the DSBs are repaired by different mechanisms, such as inter sister repair, RAD-51 and BRC-1 dependent pathways or by mechanism RAD-51 independent such as NHEJ and SSA^23–26^. This suggests a role of FCD-2 in these pathways.

In wild type animals, localization of FCD-2 and RAD-51 was spatially separated, as the former peaked only after most of the latter had disappeared, indicating that the two proteins are required at different steps during DSB processing or loaded onto different intermediates. This is further supported by the fact that RAD-51 foci are still formed in absence of *fcd-2*^17^ and vice versa. In line with these data, in absence of FCD-2, RAD-51 foci notably increased indicating that DSBs are most likely channeled into a RAD-51-mediated pathway^17,18^ Fig. 1d). This supports a different role of proteins at DSB.

Surprisingly, we also found that ectopic DSB induction rescues FCD-2 recruitment in the *spo-11* mutants, as well as triggers its loading in nuclei undergoing mitosis, confirming that it is not *spo-11* activity *per se* that is essential for FCD-2 localization but rather the presence of DSB. Growing evidence shows that FANCD2 is capable of binding ssDNA^55,56^ raising the fascinating possibility that during meiosis in worms, FCD-2 may be coating stretches of ssDNA generated upon resection, regardless of strand-invasion proficiency, as demonstrated by the fact that FCD-2 still loads when *rad-51* is not functional. Recent results obtained in mitotic cells support a direct role of FA factors in DSBs processing by promoting single strand repair: i) FANCD2 processes Cas9-induced DSBs by dictating the balance between NHEJ and single-stranded templates repair^16^ ii) the FANCA core complex protein promotes DSB repair by catalyzing bidirectional single-strand annealing (SSA) and strand exchange^57^. Our data support a role for FCD-2 in break protection/signaling during meiosis. FCD-2 may be recruited at chromosome after break formation probably in the regions flanking the break sites, where it may dictate the balance between NHEJ and homology mediated RAD-51-independent pathway such as SSA^24–26^. Another scenario may invoke localization along ssDNA regions formed upon formation of double Holliday Junction during homology-directed repair, which would be also compatible with the burst of expression of FCD-2 after early pachytene stage and its decline when pro-CO factors start to be loaded.

We also analyzed the genetic interaction between FCD-2 and BRC-1, as the latter has been shown to intersect the FA pathway in mammals. Localization of both proteins is mutually independent however contemporary lack of FCD-2 and BRC-1 leads to a synergistic effect on apoptotic cell death, indicating abrogation of at least two independent pathways. Recent works have shown that lack of *brc-1* alters RAD-51 loading dynamics resulting in a detrimental effect on its abundance^30,32,58^. In contrast, a compromised *fcd-2* function induces higher levels of RAD-51 protein. These data further support that FCD-2 and BRC-1 differently impact on DSB repair during meiosis by promoting intervention of different pathways. Further studies will hopefully further clarify the exact role of FANCD2 protein after recruitment at DNA breaks during meiosis and the relationships with its mitotic function in order to provide new insight on Fanconi Anaemia pathway. Furthermore, our transgenic mutant could be used to analyze the genetic interaction with other meiotic genes and how they are related to FA pathway during gametogenesis, in order to find new target for clinical applications.

## Supplementary information

S.I. (Figures and tables).
