# Supplementary material for: In vivo analysis of FANCD2 recruitment at meiotic DNA breaks in Caenorhabditis elegans
Source: Sci Rep. 2020 Jan 9;10:103. doi: 10.1038/s41598-019-57096-1 (PMC6952437; doi:10.1038/s41598-019-57096-1)
Supplement: Supplementary file 1 — S.I. (Figures and tables). [file 41598_2019_57096_MOESM1_ESM.pdf]

Supplementary information

In vivo analysis of FANCD2 recruitment at meiotic DNA breaks in *Caenorhabditis elegans*

Marcello Germoglio[1], Anna Valenti[1], Ines Gallo[1], Chiara Forenza[1], Pamela Santonicola[1], Nicola Silva[2], Adele Adamo[1,*]

Figure S1

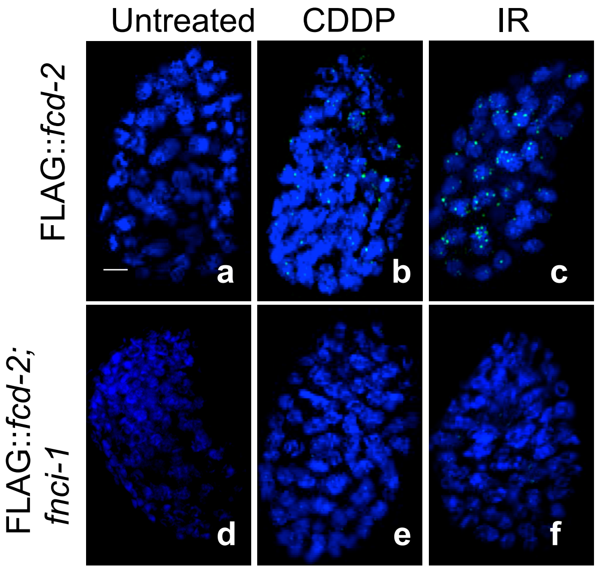

Figure S1: FCD-2 is recruited to the chromosomes after DNA damages induction in *C. elegans* embryos in FNCI-1 dependent manner.

Immunostaining with anti-FLAG antibody (green), and DAPI (blue) of *C. elegans* embryos untreated (**a**, **d**), after 48 hours treatment with180 μM CDDP (**b**,**e**) and after 1 hour 120 Gy γ-rays exposure (**c**, **f**), in *FLAG::fcd-2* and *FLAG::fcd-2; fnci-1*. Bar, 10 μm.

Supplementary information

In vivo analysis of FANCD2 recruitment at meiotic DNA breaks in *Caenorhabditis elegans*

Marcello Germoglio[1], Anna Valenti[1], Ines Gallo[1], Chiara Forenza[1], Pamela Santonicola[1], Nicola Silva[2], Adele Adamo[1,*]

Figure S2

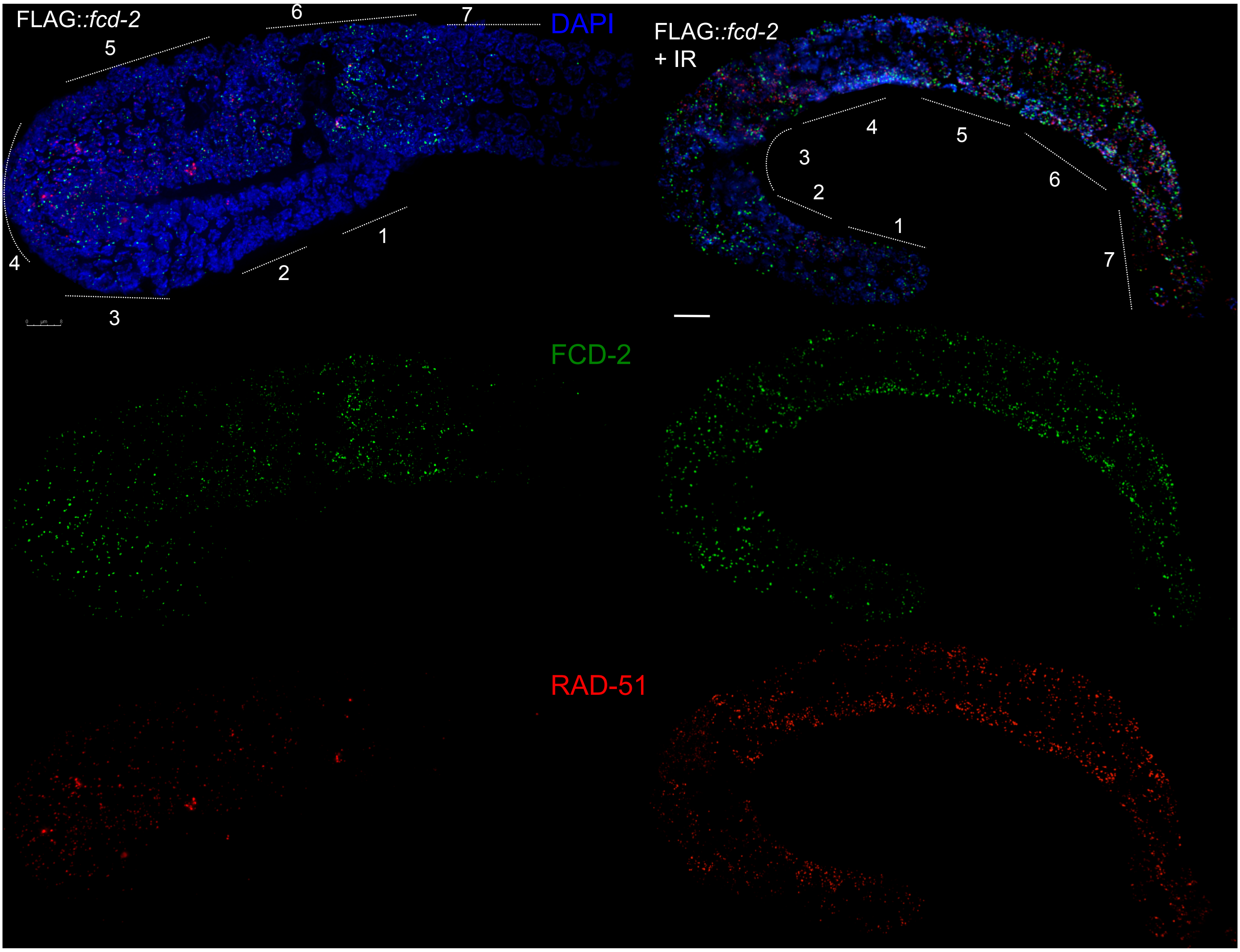

Figure S2: Localization of FLAG::FCD-2 before and after γ-rays exposure in *C. elegans* germline.

Representative image of gonad dissected from *FLAG::fcd-2* transgenic hermaphrodite worm and co-stained with anti-FLAG antibody (green), anti-RAD-51 (in red) and DAPI (blue) before and after 1 hour 120 Gy γ-rays exposure Bar, 8 μm.

Supplementary information

In vivo analysis of FANCD2 recruitment at meiotic DNA breaks in *Caenorhabditis elegans*

Marcello Germoglio[1], Anna Valenti[1], Ines Gallo[1], Chiara Forenza[1], Pamela Santonicola[1], Nicola Silva[2], Adele Adamo[1,*]

***brc-1*(tm1145)III** Tm=59°C	
Upper:	TGTCGCATCGTCGGCATTAA
Lower:	AATATAGGCACCGGCGGGGA
***fcd-2*(tm1298)IV** Tm=52°C	
Upper	TCGCTCCGCCCTCTTTTCTA
Lower	CGACGAGCAGCTAACAACATTGG
**3xFLAG::*fcd-2* IV** Tm=58°C	
Upper	TTCCCGCCATGGAGCATG
Lower	GTGGCCATCATCAGGATC
***fnci-1*(tm3081)II** Tm=58°C	
Upper	ATGATCGTGGAGGCGGAATG
Lower	GTGATCAAACTGTCCACCTC
***brd-1*(dw1)III** Tm=56°C	
Upper	ACCTGATGCGGTCTATTTGG
Lower	ATTGCGACCTGATTTGAAGG

Table S1. **List of all primers used in this study**

Supplementary information

In vivo analysis of FANCD2 recruitment at meiotic DNA breaks in *Caenorhabditis elegans*

Marcello Germoglio[1], Anna Valenti[1], Ines Gallo[1], Chiara Forenza[1], Pamela Santonicola[1], Nicola Silva[2], Adele Adamo[1,*]

Average of foci/nucleus	Mitotic zone	Early pachytene	Middle pachytene	Late pachytene
- CDDP	0.1	3	16	22
+ CDDP	9	19	>40	>40

Table S2. **Average of FLAG::FCD-2 foci per nucleus in *FLAG::fcd-2* strain, before and after 48 hours of CDDP treatment.**
An average of 60 nuclei for each gonad region were scored for each genotype.
